# 5-ALA/Fe^2+^ attenuates testicular inflammatory injury and spermatogenic failure in non-obstructive azoospermia mice by suppressing the TP53-CASP3 apoptotic cascade

**DOI:** 10.3389/fcell.2026.1884004

**Published:** 2026-07-16

**Authors:** Jiewen Zhu, Jing Zhai, Zhanxin Xu, Yifan Wang, Jinle Xie, Yangyun Wang, Jiangang Hou, Ke Xu

**Affiliations:** 1 Department of Urology, Shanghai Fifth People’s Hospital, Fudan University, Shanghai, China; 2 Department of Pelvic Floor Center, Shanghai Fifth People’s Hospital Affiliated to Fudan University, Minhang District Pelvic Floor Center, Shanghai, China; 3 Department of Urology, Huashan Hospital, Fudan University, Shanghai, China

**Keywords:** 5-ala, apoptosis, busulfan, Caspase3, non-obstructive azoospermia, TP53

## Abstract

**Background:**

Non-obstructive azoospermia (NOA) remains a major challenge in the management of male infertility, with currently limited effective therapeutic options available. The study investigates the therapeutic potential and underlying mechanism of 5-aminolevulinic acid with ferrous iron (5-ALA/Fe^2+^) for NOA.

**Methods:**

A busulfan-induced NOA mouse model was established and treated with low or high-dose 5-ALA/Fe^2+^. Therapeutic effects were assessed via testicular histopathology, hormone assays, and evaluation of apoptosis. The mechanism was systematically explored through integrated network pharmacology, proteomic analysis, and experimental validation using Western blot, qPCR, and flow cytometry.

**Results:**

5-ALA/Fe^2+^ administration significantly ameliorated testicular atrophy, restored spermatogenic architecture, and improved hormonal profiles in NOA mice. It notably upregulated Sertoli cell-specific markers (WT1, GATA4, SOX9) and suppressed testicular cell apoptosis. Integrated analysis identified the TP53-CASP3 axis as a key downstream node. Both bioinformatics predictions and experimental validation confirmed that 5-ALA/Fe^2+^ exerts its protective effect by inhibiting the TP53-CASP3 pathway, evidenced by reduced TP53 and cleaved Caspase-3 levels, decreased Bax/Bcl-2 ratio, and improved mitochondrial membrane potential and cell viability.

**Conclusion:**

Our findings demonstrate that 5-ALA/Fe^2+^ is a promising therapeutic agent for NOA, capable of mitigating testicular damage and restoring spermatogenic function. The protective effect is primarily mediated through the targeted suppression of the TP53-CASP3 apoptotic signaling pathway.

## 1 Introduction

Infertility, defined as the failure to conceive after 12 months of regular unprotected intercourse, is a significant global health challenge, affecting approximately 15% of couples (Zegers-Hochsch et al., 2017; Choy and Amory, 2020).

In approximately 50% of infertility cases, male factors are identified as a primary or contributing cause (Agarwal et al., 2021). According to the present researches, the main causes closely related to male infertility include unilateral/bilateral varicocele, gonadal axis hormone disorders, reproductive tract obstruction or absence, genetic factors, and reproductive malnutrition. These causes ultimately lead to a decrease in sperm count and vitality, as well as damage to spermatogenic function, which is the main pathogenic factor of male infertility (Zhao et al., 2021). Among the many diseases that cause male infertility, non-obstructive azoospermia (NOA) is the most severe type. NOA refers to the complete absence of sperm in the ejaculate following two or more separate semen analyses with centrifugation after the exclusion of obstructive factors. It is characterized by intrinsic testicular failure and represents the most prevalent form of azoospermia, accounting for up to 70% of all cases (Wang et al., 2025).

The etiology of NOA is highly heterogeneous, which is broadly categorized into pretesticular and testicular origins (Hubbard et al., 2025; Agarwal et al., 2020). Pretesticular causes primarily arise from dysregulation of the hypothalamic-pituitary-gonadal axis, encompassing pituitary disorders (e.g., cranial trauma, pituitary adenomas), pharmacological influences (e.g., chronic use of psychotropic agents, opioid abuse), and genetic defects such as Kallmann and Prader-Willi syndromes. Testicular causes directly involve the testes themselves, with common factors including infections, trauma, cryptorchidism, varicocele, and genetic etiologies (e.g., Klinefelter syndrome, Y-chromosome AZF microdeletions). These diverse pathogenic factors ultimately lead to severe impairment of spermatogenesis, which histologically manifests predominantly in three patterns: hypospermatogenesis, maturation arrest, and Sertoli cell-only syndrome (Mostafa et al., 2025). Notably, despite comprehensive diagnostic workups, the majority of NOA cases are classified as idiopathic, while acquired factors account for approximately 15%–21% (Punab et al., 2017; Achermann and Esteves, 2023; Tang et al., 2022).

This etiological complexity directly translates into considerable challenges in clinical management, which lacks standardized protocols. Current strategies primarily involve either empirical hormonal therapies (Alkandari and Zini, 2021) or surgical intervention, typically testicular sperm extraction (TESE) followed by intracytoplasmic sperm injection (ICSI) (Majzoub et al., 2024). Although isolated successful pregnancies have been achieved, these approaches offer limited efficacy for most patients. Critically, they do not address the underlying pathology; hormonal modulation and surgery cannot reverse the core testicular dysfunction and may, in some cases, risk exacerbating it (Ghanami Gashti et al., 2021). The therapeutic bottleneck stems from two fundamental hurdles: (1) the multifactorial pathophysiology involving hormonal axis dysregulation, oxidative stress, and genetic abnormalities; and (2) the unique immunological and physiological microenvironment of the seminiferous epithelium, which hampers targeted drug delivery (Chen et al., 2023). Hence, it is urgent and imperative to gain an in-depth understanding of the pathophysiological mechanisms underlying NOA and to identify novel therapeutic targets or ideal agents.

Busulfan (1,4-butanediol dimethanesulfonate) is an alkylating chemotherapeutic agent commonly employed in the treatment of myeloproliferative syndromes, chronic myeloid leukemia, lymphoma, and ovarian cancer (Mobarak et al., 2022). Beyond its therapeutic applications, busulfan is associated with a range of adverse effects, including significant disruption of the reproductive system (Li et al., 2023). Busulfan exerts its cytotoxic effects primarily through the formation of DNA–DNA cross-links, DNA–protein cross-links, and single-strand breaks. In the adult testis, it mainly targets cells in the G1 phase (Feng et al., 2024). Besides, studies have shown that busulfan can induce orchitis by elevating cytokine levels, which disrupts the blood-testis barrier, compromises spermatogenic function, and leads to extensive apoptosis of spermatogenic cells (Zhao et al., 2023; de Kloet et al., 2022). Due to its sustained suppressive effect on spermatogenesis, busulfan is widely used in preclinical research to induce reliable NOA animal models.

5-Animolevulinic acid (5-ALA) is an endogenous amino acid widely distributed in plants, animals, and microorganisms. As a crucial precursor for the biosynthesis of tetrapyrrole compounds such as heme and vitamin B12, it plays fundamental roles not only in basic physiological processes but also centrally regulates immune-inflammatory responses, oxidative stress, mitochondrial function, and metabolism (Ebrahimi et al., 2024). The classical application of 5-ALA lies in photodynamic therapy (PDT): following cellular uptake, exogenous 5-ALA is metabolized via the endogenous heme synthesis pathway into protoporphyrin IX (PpIX), a highly photosensitive intermediate. Upon irradiation with light of a specific wavelength, PpIX generates cytotoxic reactive oxygen species, predominantly singlet oxygen, leading to targeted tumor cell death (Tan et al., 2015). 5-ALA/Fe^2+^ has been reported to exert anti-inflammatory and antioxidant effects in various disease models (Hou et al., 2013; Nishio et al., 2014; Hou et al., 2015). Additionally, it can modulate the expression of key metabolic regulators such as PGC-1α, COX-IV, UCP1/2, and GLUT1/2 (Kuryata et al., 2024). Our previous research has confirmed that 5-ALA/Fe^2+^ could ameliorate testicular heat stress injury in mice, involving inhibition of MAPK-mediated oxidative stress and apoptosis pathways (Gao et al., 2023).

On this basis, we attempted to further explore whether 5ALA/Fe^2+^ has potential therapeutic effects on NOA. Using busulfan-induced mouse models of NOA, we demonstrated that the administration of 5-ALA/Fe^2+^ significantly improved testicular parameters. Through integrated proteomics, network pharmacology analysis, and a series of functional validation experiments, we identified that the therapeutic mechanism of 5-ALA/Fe^2+^ involves selective modulation of the TP53-CASP3 apoptosis axis, suggesting a targeted approach for spermatogenic recovery.

## 2 Materials and methods

### 2.1 Animals

Forty male mice (6–8 weeks old with an average weight of 30 g) were provided by the CHEDUN Experimental Animal Seed Multiplication Farm of Shanghai and housed in the standard conditions (22 °C ± 3 °C,40%–60% humidity) with 12 h light/dark cycles and unlimited access to water and chewing foods. All procedures involving animals were performed in compliance with the Guide for the Care and Use of Laboratory Animals (Ministry of Science and Technology of China, 2006). The experimental protocol was reviewed and approved by the Institutional Animal Care and Use Committee (IACUC) of the Department of Laboratory Animal Science, Fudan University (Approval No. 2017-1325-A253).

### 2.2 Reagent preparation

Busulfan was purchased from Sigma-Aldrich (Batch No. B2635). The 5-ALA/HCl (Merck Millipore, United States) and Fe^2+^ (sodium ferrous citrate, Fanhai Biotechnology, China) were prepared as described previously (Hou et al., 2013). Briefly, both compounds were dissolved in distilled water at a molar ratio of 1:0.5 (5-ALA to Fe^2+^). The Fe^2+^ solution was diluted in distilled water immediately prior to each administration, and light exposure was minimized throughout the process. The 5-ALA/Fe^2+^ mixture was administered orally in a volume of 0.3 mL of distilled water.

### 2.3 Animal study design

A mouse model of NOA was established by a single intraperitoneal injection of busulfan at a dose of 40 mg/kg, in accordance with previously established protocols (Ziaeipour et al., 2019; Rezaei et al., 2021; Pu et al., 2023). The sample size of 10 mice per group was determined empirically based on our preliminary experiments and previously published studies using busulfan-induced NOA models (Hou et al., 2013; Hou et al., 2015).

After 1 week of acclimatization, the mice were randomly assigned into four groups (n = 10 per group) using a random number table:

1. Control group: Healthy mice without any treatment (double-distilled water as placebo).
2. NOA group: Mice subjected to the busulfan-induced NOA model (double-distilled water as placebo).
3. NOA + Low-dose 5-ALA/Fe^2+^: NOA mice treated with a low dose of 5-ALA/Fe^2+^ (30 mg/kg).
4. NOA + High-dose 5-ALA/Fe^2+^: NOA mice treated with a high dose of 5-ALA/Fe^2+^(100 mg/kg).

The therapeutic interventions were started 1 month after busulfan injection. It has been established that a single busulfan injection (40 mg/kg) leads to stable and nearly complete depletion of endogenous spermatogenic cells by 4 weeks, making this time point suitable for treatment initiation in NOA models (Ziaeipour et al., 2019; Rezaei et al., 2021; Pu et al., 2023).

At the start of treatment (1 month after busulfan), body weights of the three busulfan-exposed groups (NOA, NOA + low-dose, NOA + high-dose) were comparable (p > 0.05), confirming group equivalence for inter-group comparison. All treatments were administered orally by gavage once daily for four consecutive weeks. During the 4-week treatment period, no animals were lost. The general condition of the mice in all groups was closely monitored and recorded throughout the study. Twenty-four hours after the final administration, mice were anesthetized by intraperitoneal injection of 1% pentobarbital sodium. Approximately 100 μL of blood was collected from each mouse and placed into sodium citrate-containing tubes (blue cap). The tubes were temporarily stored at 4 °C and centrifuged within 1 h at 3500 rpm for 10 min. The resulting supernatant (plasma) was carefully transferred to 2 mL microcentrifuge tubes and stored at −20 °C for subsequent biochemical measurements.

Then all mice were euthanized by cervical dislocation. Following a midline abdominal incision, the left and right testes along with the epididymides were carefully dissected and weighed. The epididymides and one-half of the left testis were fixed for subsequent histological sectioning. Major organs, including the heart, liver, spleen, and kidneys, were also harvested from the mice for subsequent histological staining. The remaining half of the left testis was used for the isolation of Sertoli cells (SCs), while the right testis was snap-frozen for subsequent protein extraction and analysis.

For histological scoring and ImageJ-based quantification of TUNEL and PCNA staining, the investigator was blinded to group allocation. Slides were coded and analyzed in a randomized order.

### 2.4 Body and testis weight

Prior to sacrifice, all mice were weighed. Following dissection, the testes were carefully isolated from the surrounding adipose tissue, and their weights were recorded. The testis index (relative testis weight) was then calculated for each animal according to the formula as follows:

$$\text{Testis index}=\left[\text{Testis weight }\left(g\right)\;/\text{ Body weight }\left(g\right)\right]\times 100\%.$$

### 2.5 HE staining

The harvested epididymal, testicular tissues and major organs (heart, liver, spleen, and kidneys) were fixed in 4% paraformaldehyde for 24 h. Subsequently, the fixative was washed off with distilled water, and the tissues were dehydrated through a graded ethanol series, cleared in xylene, and embedded in paraffin. Sections were cut at a thickness of 5 μm and dried in an oven at 50 °C. Following deparaffinization with xylene and a reverse ethanol gradient, the sections were stained with hematoxylin for 15 min, differentiated in acid ethanol for 35 s, and counterstained with eosin for 10 min. After a final differentiation in 90% ethanol for 40 s, the sections were mounted with neutral balsam and examined under a microscope.

### 2.6 Detection of important hormones

Serum levels of testosterone, luteinizing hormone (LH), and inhibin B (INHB) were quantified using commercial mouse enzyme-linked immunosorbent assay (ELISA) kits (Testosterone: AB-B5298; LH: AB-K544102; INHB: AB-B2010811; ABmart, China), following the manufacturers’ protocols. Briefly, standards and samples were added to the antibody-precoated wells and incubated. After incubation and washing, a biotin-conjugated detection antibody was added, followed by another incubation and wash cycle. Subsequently, a horseradish peroxidase (HRP)-streptavidin conjugate was added. Following a final wash step, a tetramethylbenzidine (TMB) substrate solution was added to develop color. The reaction was stopped with a stop solution, and the optical density (OD) was immediately measured at 450 nm using a microplate reader. The hormone concentration in each sample was calculated by interpolating from the respective standard curve.

### 2.7 Detection of key serum safety biomarkers

To assess the potential systemic toxicity and the impact on major organ functions following 5-ALA treatment, key serum safety biomarkers were quantified. Hemoglobin (Hb) levels were measured using the cyanmethemoglobin method to evaluate hematological toxicity. The cardiac isoenzyme of creatine kinase (CK-MB), a specific marker for cardiac injury, was analyzed. Alanine aminotransferase (ALT) and creatinine levels were determined to evaluate hepatic and renal functions, respectively. All assays were performed in duplicate using standardized commercial kits (Abbott Laboratories, United States) according to the manufacturers’ instructions on an automated biochemistry analyzer (Architect c8000, Abbott).

### 2.8 Assessment of oxidative stress levels in testicular tissues

Given the dual role of ferrous ions, the accumulation of Fe^2+^ may exacerbate oxidative stress. Therefore, to evaluate the efficacy and dose safety of 5-ALA/Fe^2+^, oxidative stress levels in mouse testicular tissues were assessed. Commercial kits for superoxide dismutase (SOD) activity and lipid peroxidation (malondialdehyde, MDA) assays (Beyotime, China) were used according to the manufacturers’ instructions. Briefly, for the SOD assay, standards and samples were added to 96-well plates, followed by the addition of WST-8/enzyme working solution and reaction initiating working solution. After incubation at 37 °C for 30 min, the optical density (OD) was measured at 450 nm, and SOD activity was calculated using the inhibition percentage. For the MDA assay, standards and samples were mixed with MDA detection working solution, heated at 100 °C for 15 min, cooled, and centrifuged. The supernatants were then transferred to 96-well plates, and the OD was measured at 532 nm. MDA concentrations were determined from a standard curve. SOD activity and MDA content in each sample were calculated using the corresponding standard curves or formulas.

### 2.9 Immunofluorescence staining on testicular tissues

Paraffin-embedded testicular sections (4–5 µm) were deparaffinized, rehydrated, and subjected to heat-induced antigen retrieval in citrate buffer (pH 6.0) at 100 °C for 20 min. After blocking with 5% normal goat serum in PBS for 1 h at room temperature, the sections were processed as follows.

For single SOX9 labeling, sections were incubated overnight at 4 °C with SOX9 antibody (1:500; Abcam, ab185230, rabbit monoclonal). After washing with PBS, the sections were incubated with Alexa Fluor® 488-conjugated goat anti-rabbit IgG (1:500; Beyotime, China) for 1 h at room temperature in the dark. Nuclei were counterstained with DAPI, and the sections were mounted with anti-fade mounting medium (Beyotime, China).

For co-staining with TUNEL or PCNA, the same anti-SOX9 antibody was used. Sections were incubated overnight at 4 °C, followed by incubation with Alexa Fluor® 488-conjugated goat anti-rabbit IgG for 1 h at room temperature in the dark. After washing with PBS, nuclei were counterstained with DAPI, and the sections were mounted. The positions of the fields of view were recorded to enable relocation after subsequent TUNEL or PCNA staining.

### 2.10 Immunofluorescence staining on cultured Sertoli cells

To identify Sertoli cells isolated from testicular tissue, immunofluorescence staining was performed on the cultured cells. The isolated testicular cells grown on coverslips were fixed with 4% paraformaldehyde for 15 min at room temperature, permeabilized with 0.1% Triton X-100 in PBS for 10 min, and blocked with 5% normal goat serum in PBS for 1 h at room temperature. For single labeling of SOX9, WT1, SCP3, CYP17A1, and HSD3β, cells were incubated overnight at 4 °C with the following primary antibodies: anti-SOX9 (1:500; Abcam, ab185230, rabbit monoclonal), anti-WT1 (1:200; Abcam, ab89901, rabbit monoclonal), anti-SCP3 (1:500; Abcam, ab15093, rabbit polyclonal), anti-CYP17A1 (1:200; Invitrogen, MA5-35632, rabbit monoclonal), and anti-HSD3β (1:500; Invitrogen, PA5-106895, rabbit polyclonal). As a negative control, PBS was used instead of the primary antibody. After washing with PBS, the cells were incubated with Alexa Fluor® 488-conjugated goat anti-rabbit IgG (1:500; Beyotime, China) for 1 h at room temperature in the dark. Nuclei were counterstained with DAPI, and the coverslips were mounted with anti-fade mounting medium (Beyotime, China). Fluorescence images were captured using a microscope.

### 2.11 TUNEL staining

To detect the presence of apoptotic cells in testicular tissues, the Terminal deoxynucleotidyl transferase-mediated dUTP nick end labeling (TUNEL) assay was performed using a commercial kit (Beyotime, China) following the manufacturer’s protocol. Testes from 10 mice per group were used, and one testis per mouse was analyzed. After the staining procedure, sections were examined under a microscope, and images of TUNEL-positive signals were captured. For each testis, three randomly selected fields were analyzed, and the percentage of TUNEL-positive cells was quantified using ImageJ software. For statistical analysis, the percentage of TUNEL-positive cells per testis was calculated as the mean of the three fields, and the per-animal values (n = 10 per group) were used for group comparisons.

### 2.12 PCNA staining

To detect proliferating cells in testicular tissues, immunofluorescence staining for proliferating cell nuclear antigen (PCNA) was performed using an anti-PCNA antibody (Abcam, UK). Briefly, sections were incubated with the primary antibody overnight at 4 °C, washed, and then incubated with a fluorescence-conjugated secondary antibody. After counterstaining with DAPI, sections were examined under a fluorescence microscope, and images of PCNA-positive cells were captured. Testes from 10 mice per group were used, and one testis per mouse was analyzed. For each testis, three randomly selected fields were analyzed, and the percentage of PCNA-positive cells was quantified using ImageJ software. For statistical analysis, the percentage of PCNA-positive cells per testis was calculated as the mean of the three fields, and the per-animal values (n = 10 per group) were used for group comparisons.

### 2.13 Western blot analysis

The expression levels of Sertoli cell-specific proteins (GATA4 and WT1) and key apoptosis-related molecules (Bax, Bcl-2, and cleaved caspase-3) in testicular tissue were analyzed by Western blot. Testicular tissues from all 10 mice per group were used for Western blot analysis. Previous studies have reported ACTB (β-actin) as a reliable loading control in testicular pathological models (Wang et al., 2024; Wang et al., 2023). It is stably expressed across major testicular somatic cell types, including Sertoli and Leydig cells. We have confirmed that β-actin bands showed stable expression across all groups in this study. Therefore, β-actin was selected as the loading control for our Western blotting analysis. Testicular tissues were homogenized in lysis buffer, and protein concentrations were determined using the BCA assay. Proteins were separated by SDS-PAGE and transferred to PVDF membranes. After blocking with 5% non-fat milk, the membranes were incubated overnight at 4 °C with the following primary antibodies: β-Actin (1:1000; Abcam, ab8226, mouse monoclonal), Bax (1:1000; CST, 2772S, rabbit monoclonal), Bcl-2 (1:1000; CST, 3498S, rabbit monoclonal), cleaved caspase-3 (1:1000; CST, 9661S, rabbit polyclonal), caspase-3 (1:1000; CST, 9662S, rabbit monoclonal), GATA4 (1:500; Santa Cruz, sc-25310, mouse monoclonal), WT1 (1:1000; Abcam, ab89901, rabbit monoclonal), TP53 (1:5000; Proteintech, 60283-2-Ig, mouse monoclonal), PTGS2 (1:1000; Proteintech, 12375-1-AP, rabbit polyclonal), and MAPK3 (1:5000; Proteintech, 11257-1-AP, rabbit polyclonal). Following incubation with appropriate HRP-conjugated secondary antibodies at room temperature for 1 h, the protein bands were visualized using an ECL substrate (Merck, Germany) and quantified with ImageJ software.

### 2.14 Extraction, isolation, and culture of Sertoli cells

Based on previously established and optimized protocols, this study employed a three-step enzymatic digestion method to isolate highly purified testicular SCs (Gerber et al., 2020; Nenicu et al., 2007; Saewu et al., 2020). The decapsulated testicular tissues were sequentially digested with three specifically formulated media: Digestive medium 1 (1 mg/mL collagenase +20 μg/mL DNase), Digestive medium 2 (2 mg/mL collagenase +20 μg/mL DNase +2 mg/mL hyaluronidase), and Digestive medium 3 (identical composition to medium 2). Each digestion was performed at 37 °C with continuous shaking, followed by gravity sedimentation or brief centrifugation at 50-100 × g. After the final digestion, cells were filtered through a 70-μm strainer and seeded at 50,000 cells/cm^2^ in complete medium (DMEM/Ham’s F12 + 10% FBS). To eliminate residual germ cells, a 5-min hypotonic treatment with 20 mM Tris-HCl (pH 7.5) was applied on day 3 of culture. The purified SCs were maintained for an additional 24 h before experimental use.

To assess the purity of isolated SCs, immunofluorescence staining for WT1 or SOX9 was performed as described in Section 2.10. For each coverslip, six random fields were captured, and the percentages of WT1-positive or SOX9-positive nuclei among DAPI-positive nuclei were independently estimated by two researchers. Additionally, the absence of contaminating cell types was assessed by staining for SCP3, CYP17A1, and HSD3β. No positive staining for these markers was observed.

### 2.15 Flow cytometry analysis

Apoptosis was detected using an Annexin V-FITC staining kit, allowing for the quantification of phosphatidylserine externalization on the cell surface. Sertoli cells isolated from each of the 10 mice per group were analyzed individually. Simultaneously, alterations in ΔΨm were evaluated with the fluorescent probe JC-1, which exhibits a potential-dependent shift in fluorescence emission from red (aggregates in healthy mitochondria) to green (monomers in depolarized mitochondria). Briefly, isolated SCs were resuspended in appropriate assay buffers. For the apoptosis assay, cells were stained with Annexin V-FITC. For the ΔΨm assay, cells were loaded with JC-1 dye and incubated at 37 °C. After the incubation periods, cells were analyzed immediately by flow cytometry. The fluorescence of Annexin V-FITC was detected in the FL1 channel, while JC-1 monomers and aggregates were detected in the FL1 and FL2 channels, respectively. Carbonyl cyanide m-chlorophenyl hydrazone (CCCP)-treated cells were used as a positive control for mitochondrial depolarization in the JC-1 assay. Data were processed through FlowJo software to determine the percentage of apoptotic cells and the ratio of red/green fluorescence, which is indicative of ΔΨm status.

### 2.16 Cell viability assay

Cell viability was assessed using the Cell Counting Kit-8 (CCK-8) according to the manufacturer’s instructions. Briefly, SCs were seeded in a 96-well plate at a density of 5 × 10^3^ cells per well and cultured for 24 h. After treatment, 10 µL of CCK-8 solution was added to each well, followed by incubation at 37 °C for 1–4 h. The absorbance was measured at 450 nm using a microplate reader. Cell viability was expressed as a percentage relative to the control group.

### 2.17 Quantitative real-time PCR

Total RNA was extracted from testicular tissues using the Total RNA Extraction Kit (Promega, WI, United States) according to the manufacturer’s instructions. RNA concentration and purity were determined spectrophotometrically (OD260/OD280 ratio between 1.9 and 2.1). cDNA was synthesized from 1 μg of total RNA using a reverse transcription kit (PrimeScript™ RT reagent Kit with gDNA Eraser). Ten animals per group were analyzed as biological replicates, each with three technical replicates. The mRNA expression levels of TP53, PTGS2, MAPK3 and Caspase-3 were analyzed by qPCR using SYBR Green Master Mix on a Real-Time PCR System. The reaction conditions were as follows: initial denaturation at 95 °C for 30 s, followed by 40 cycles of 95 °C for 5 s and 60 °C for 30 s. A melt curve analysis was performed from 60 °C to 95 °C to verify amplicon specificity; each primer pair produced a single peak, confirming the absence of primer-dimers and non-specific amplification. Primer sequences, amplicon lengths, and Tm values are provided in Supplementary Table S1. The β-actin gene was used as an internal control for normalization, and relative gene expression was calculated using the 2^(-ΔΔCt)^ method.

### 2.18 Network pharmacology analysis

Potential targets of 5-aminolevulinic acid (5-ALA) and busulfan were predicted using multiple bioinformatics databases. The SMILES and SDF structures of both compounds were obtained from PubChem and submitted to SwissTargetPrediction (based on ChEMBL 23, accessed 13 June 2025 for 5-ALA and 16 June 2025 for busulfan), PharmMapper (2017 update, accessed 15 June 2025 for 5-ALA and 17 June 2025 for busulfan), and TargetNet (latest version, accessed 17 June 2025 for both) for target prediction with a probability threshold >0. Additional targets were retrieved from the CTD database (v1.1, accessed 13 June 2025 for busulfan and 17 June 2025 for 5-ALA). All predicted targets were standardized using UniProt (ID mapping tool, accessed 17 June 2025) and consolidated into non-redundant datasets, yielding 449 targets for 5-ALA and 285 for busulfan. Disease-related targets for NOA were collected from GeneCards (version 5.24, searched 15 June 2025), OMIM (Gene Map Retrieval, accessed 15 June 2025), DisGeNET (version 7.0, accessed 16 June 2025), and CTD (v1.1, accessed 16 June 2025). A Venn analysis was performed to identify overlapping targets among 5-ALA, busulfan, and NOA using the R package ggvenn (version 0.1.10). The common targets were then used to construct a protein-protein interaction (PPI) network via the STRING database (version 12.5, accessed June 2025), with the species set as “*Homo sapiens*” and a minimum interaction score of 0.4. Functional enrichment analysis, including Gene Ontology (GO) and Kyoto Encyclopedia of Genes and Genomes (KEGG) pathway analyses (KEGG release 110.0, accessed 16 June 2025), was conducted on the overlapping targets using the R packages org. Hs.e.g.,.db (version 3.20.0) and clusterProfiler (version 4.18.3).

### 2.19 Proteomic analysis

Proteins were extracted from each sample using SDT lysis buffer. Protein concentration was determined by the BCA assay, and 15 µg of protein from each sample was separated by SDS-PAGE for quality control (Hu et al., 2025). A pooled QC sample was prepared by combining equal amounts of protein from all samples. Proteins were digested using the filter-aided proteome preparation (FASP) method with trypsin. The resulting peptides were desalted using a C18 cartridge, lyophilized, and reconstituted in 0.1% formic acid. Peptide concentration was measured by OD_280_, and iRT standards were added prior to data-independent acquisition (DIA) analysis on an Astral mass spectrometer. Peptides were separated by nanoflow liquid chromatography (Vanquish Neo, Thermo Scientific) and analyzed using an Astral mass spectrometer in DIA mode. The mass range was set to 380–980 m/z, with MS^1^ and MS^2^ resolutions of 240,000 and variable isolation windows, respectively. DIA data were processed using DIA-NN software, allowing for one missed cleavage, fixed carbamidomethylation, and variable modifications (oxidation and N-terminal acetylation), at an FDR threshold <1%. Six biological replicates per group were analyzed. A total of 10,057 proteins were quantified across all samples. No additional protein-level FDR correction was applied. Principal component analysis (PCA) was performed in R software after Pareto scaling. Functional annotation was performed using Blast2GO (version 6.0) for Gene Ontology (GO) and KOBAS (version 3.0) for KEGG pathway analysis. Enrichment analysis was conducted using Fisher’s exact test.

### 2.20 Statistical analysis

Data are presented as means ± SD. For continuous variables (e.g., body weight, testis index, hormone levels, Western blot band intensity, qPCR, flow cytometry data, and cell viability), comparisons among multiple groups were performed using one-way analysis of variance (ANOVA) followed by Tukey’s multiple comparison test. For the ordinal Johnsen scores, the Kruskal-Wallis test followed by Dunn’s post hoc test was used. A P-value <0.05 was considered statistically significant. Statistical analyses were performed using GraphPad Prism 9.0.0 (GraphPad Software, United States). Image quantification was performed using Fiji (ImageJ) software.

## 3 Results

### 3.1 Effects of 5-ALA/Fe^2+^ on body weight in busulfan-induced NOA mice

**FIGURE 1:**
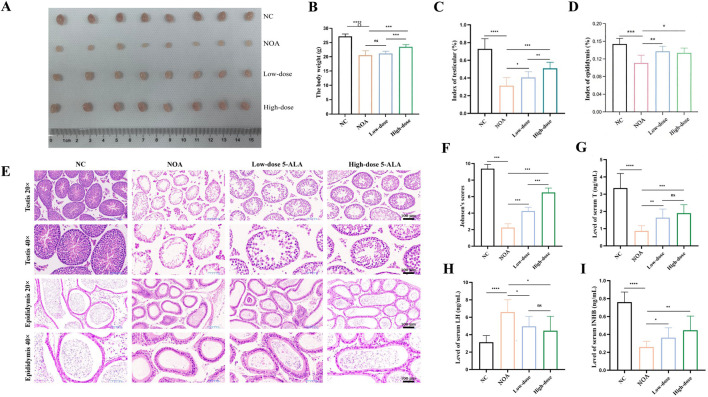
5-ALA/Fe2+ ameliorates the NOA phenotype. **(A)** Testicular morphology. **(B)** Body weight dynamics. **(C,D)** Testicular and epididymis indices. **(E)** Histopathological analysis of testis and epididymis (scale bar = 100 μm). **(F)** Johnson’s score quantification from **(E)**. **(G–I)** Serum hormone levels: **(G)** Testosterone, **(H)** Luteinizing hormone, **(I)** Inhibin **(B)**. Data are expressed as mean ± SD (n = 10 in each group). *p < 0.05, **p < 0.01, ***p < 0.001, ****p < 0.0001 versus the indicated control group; ns, not significant.

The results revealed that mice with busulfan-induced NOA exhibited a marked decrease in body weight compared to the normal control group (P < 0.0001). Treatment with 5-ALA/Fe^2+^ resulted in an increase in average body weight across the treatment groups. Notably, the high-dose 5-ALA group showed a significant recovery in body weight, demonstrating a statistically significant difference compared to the disease model group (P < 0.001). In contrast, although a slight increase was observed, the low-dose 5-ALA group did not show a significant difference in body weight compared to the NOA group (Figure 1B).

### 3.2 Effects of 5-ALA/Fe^2+^ on testicular and epididymal morphology in busulfan-induced NOA mice

Upon completion of the experiment, the right testes were collected from all mice in each group. Macroscopic and morphometric analyses indicated severe testicular atrophy in the NOA group compared to the normal control group. In contrast, 5-ALA/Fe^2+^ treatment resulted in a dose-dependent recovery of testicular morphology and volume. Both low- and high-dose 5-ALA treatments partially reversed the busulfan-induced testicular atrophy. Notably, the testicular size in mice receiving the high-dose 5-ALA treatment closely resembled that of the normal group based on gross morphological observation (Figure 1A).

Compared to the normal control group, busulfan-induced NOA mice exhibited a significant decrease in the index of testicular (P < 0.0001). Administration of 5-ALA/Fe^2+^ led to a recovery of the index in both treatment groups. The low-dose group demonstrated a statistically significant improvement compared to the NOA group (P < 0.05), while the high-dose group exhibited a more pronounced increase, with a highly significant difference (P < 0.001). Moreover, the difference between the low- and high-dose groups was also statistically significant (P < 0.01), indicating that 5-ALA/Fe^2+^ ameliorates testicular parameters in a dose-dependent manner (Figure 1C).

Similarly, the index of epididymis was significantly reduced in NOA mice (P < 0.001) but showed notable recovery following 5-ALA/Fe^2+^ treatment (P < 0.05). However, unlike the testicular index, the index of epididymis did not display a clear dose-dependent response. In fact, the low-dose group even showed a slightly higher value than the high-dose group (Figure 1D).

### 3.3 Effects of 5-ALA/Fe^2+^ on testicular pathology in busulfan-induced NOA mice

Pathologically and morphologically, it could be observed that compared with the negative control group, the testes of NOA mice exhibited marked atrophy and a reduction in interstitial cells, although the seminiferous tubules retained their basic tubular structure. Within the tubules, the spermatogenic cell layers were severely diminished and disorganized, with a near absence of mature sperm. The seminiferous epithelium became apparently thinner, with reduced stratification, vacuolar degeneration, and looser cellular arrangement. Some SCs underwent degeneration, leading to the shedding of spermatogenic cells. Additionally, inflammatory cell infiltration was evident within the interstitial tissue of the testes. Consistent with these findings, the epididymal lumen also showed a pronounced decrease in sperm count, along with the presence of cellular debris and non-sperm components (Figure 1E). The Johnsen score ranged from 2 to 3 (2.25 ± 0.19). These collective findings demonstrate that busulfan administration caused severe damage to both testicular and epididymal structures, confirming the successful induction of the NOA mouse model.

In the low-dose 5-ALA group, the testicular seminiferous tubule lesions still existed, with a decrease in the spermatogenic cell layer, but the spermatogonial cell layer was clearly visible. The cell arrangement was orderly but still relatively loose. Interstitial cells were increased, although a few of inflammatory cells still persisted in the interstitium. Similarly, the damage of epididymis was also reversed to some extent. Scattered spermatogenic cells could be observed despite decreased sperm count (Figure 1E). The Johnsen score ranged from 4 to 5 (4.25 ± 0.19).

Furthermore, the testicular morphology of mice in the high-dose 5-ALA group demonstrated significant improvement, with seminiferous tubules exhibiting regular distribution and complete structure. A well-organized spermatogenic hierarchy was observed, with cells tightly arranged in stratified layers that sequentially differentiated from basal spermatogonia to luminal spermatozoa through primary/secondary spermatocytes and spermatids. Correspondingly, the epididymal lumen contained abundant spermatozoa without any detectable non-sperm cellular components (Figure 1E). The spermatogenic function showed marked recovery, as reflected by Johnsen scores ranging from 6 to 7 (6.5 ± 0.25). Statistical analysis revealed significant differences in Johnsen scores among the experimental groups (P < 0.001) (Figure 1F).

### 3.4 Effects of 5-ALA/Fe^2+^ on hormones in busulfan-induced NOA mice

Compared to the normal control group, serum levels of testosterone (T) and inhibin B (INHB) were significantly decreased in NOA mice (P < 0.0001). Treatment with 5-ALA/Fe^2+^ resulted in a dose-dependent recovery of both hormones, with the high-dose group showing more pronounced restoration relative to the NOA group (P < 0.001 for T; P < 0.01 for INHB) (Figures 1G,I).

In contrast, serum luteinizing hormone (LH) levels were markedly elevated in busulfan-induced NOA mice (P < 0.0001). Both 5-ALA/Fe^2+^ treatment groups exhibited a significant reduction in LH compared to the NOA model (P < 0.05), although no statistically significant difference was observed between the low- and high-dose groups, suggesting the absence of a clear dose-response relationship for LH (Figure 1H).

These hormonal alterations indicated that busulfan impairs testicular steroidogenesis, leading to dysregulation of the pituitary-gonadal axis and compromised reproductive function. 5-ALA/Fe^2+^ treatment appears to reverse this endocrine imbalance and promote functional recovery of testicular activity, showing a partial dose-dependent efficacy.

### 3.5 5-ALA/Fe^2+^ has lower organ and systemic toxicity

**FIGURE 2:**
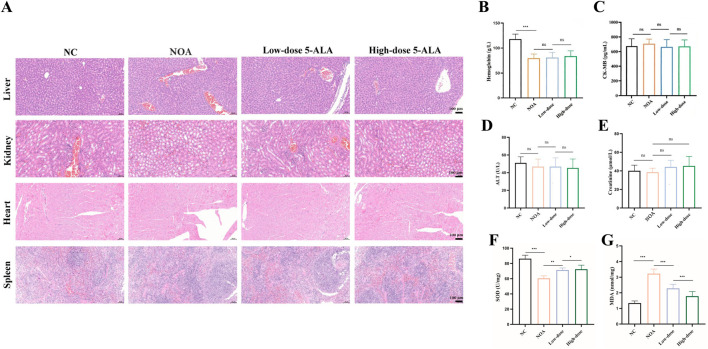
5-ALA/Fe^2+^ demonstrates a favorable safety profile. **(A)** Histopathology of major organs (liver, kidney, heart, spleen) (scale bar = 100 μm). **(B–E)** Biochemical indicators of organ function: **(B)** Hemoglobin, **(C)** CK-MB, **(D)** ALT, **(E)** Creatinine. **(F,G)** Testicular oxidative stress indicators: **(F)** SOD, **(G)** MDA. Data are expressed as mean ± SD (n = 10 in each group). *p < 0.05, **p < 0.01, ***p < 0.001, ****p < 0.0001 versus the indicated control group; ns, not significant.

HE staining revealed that the main organs (heart, liver, spleen, kidney) of all groups (normal control group, NOA model, and two 5-ALA/Fe^2+^ treatment groups) had intact structures and no abnormalities (Figure 2A). The results demonstrated that the local distribution of busulfan selectively induces testicular damage without impairing the function of other organs, confirming its viability for NOA modeling. Besides, from the perspective of histology, 5-ALA/Fe^2+^ administration exhibited no adverse effects on other vital organs, validating its systemic safety.

To comprehensively evaluate the safety of 5-ALA/Fe^2+^, blood biochemical tests were performed on all experimental mice to assess the function of vital organs, including the heart, liver, and kidneys. A significant decrease in hemoglobin was observed in all busulfan-induced NOA model groups (both untreated and 5-ALA/Fe^2+^-treated) compared to the normal controls, which was attributable to the known toxicity of busulfan (Figure 2B). Crucially, no significant differences were found in the levels of organ-specific functional markers—CK-MB (heart), ALT (liver), and creatinine (kidneys)—across any of the groups (Figures 2C–E). These findings suggest that 5-ALA/Fe^2+^ treatment exhibits a favorable safety profile, without impairing the function of these vital organs.

### 3.6 Effects of 5-ALA/Fe^2+^ on testicular oxidative stress levels in busulfan-induced NOA mice

To further assess the injury status of the busulfan-induced NOA models and comprehensively evaluate the safety of the two doses of 5-ALA/Fe^2+^ used in this study, the levels of superoxide dismutase (SOD) and malondialdehyde (MDA) in mouse testicular tissues were measured. The results showed that, compared with the normal control group, the NOA model group exhibited a significant decrease in SOD levels (P < 0.001) and a significant increase in MDA levels (P < 0.001), indicating that busulfan successfully induced testicular oxidative stress, characterized by reduced antioxidant capacity and exacerbated lipid peroxidation. After treatment with two different doses of 5-ALA/Fe^2+^, SOD levels were restored to varying degrees, while MDA levels were decreased accordingly. Moreover, the high-dose group showed significantly greater improvements than the low-dose group (SOD: P < 0.05; MDA: P < 0.001) (Figures 2F,G).

These findings suggest that 5-ALA/Fe^2+^ treatment effectively alleviates busulfan-induced testicular oxidative stress in a dose-dependent manner and exhibits good safety within the dose range used in this study, potentially exerting protective effects on testicular tissue by enhancing antioxidant capacity and inhibiting lipid peroxidation.

### 3.7 Network pharmacology analysis of the potential protective mechanism of 5-ALA

**FIGURE 3:**
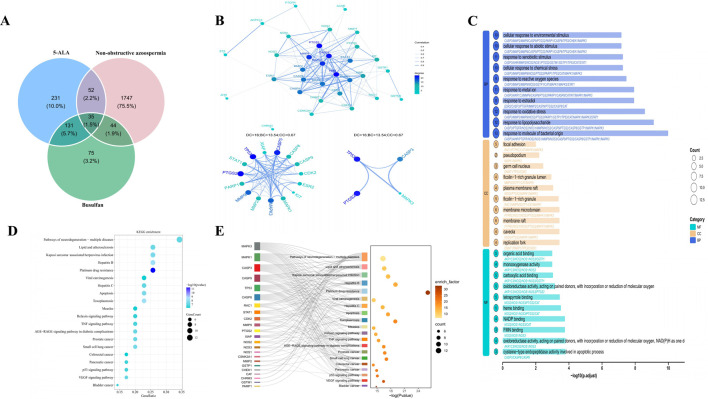
Network pharmacology analysis of the potential protective mechanism of 5-ALA. **(A)** Venn diagram of potential targets from 5-ALA, busulfan and NOA datasets. **(B)** PPI network construction and core target identification. **(C)** GO enrichment analysis. **(D)** KEGG pathway enrichment analysis. **(E)** Sankey diagram of target-pathway relationships.

In order to further explore the therapeutic effects of 5-ALA, a network pharmacology analysis of NOA, busulfan, and 5-ALA was performed, aiming to predict the potential targets and pathways of 5-ALA. Integrating the SwissTargetPrediction, TargetNet, PharmMapper, and Comparative Toxicogenomics Database (CTD) resources, we identified 449 putative targets for 5-ALA and 285 for busulfan. Concurrently, 1,878 NOA-related targets were retrieved from GeneCards, OMIM, DisGeNET, and CTD. Subsequent Venn diagram analysis of these three target sets revealed 35 overlapping genes (Figure 3A).

#### 3.7.1 PPI construction and key target discovery

A protein-protein interaction (PPI) network was then constructed using interaction data for these intersecting targets retrieved from the STRING database. This network comprised 32 nodes and 176 edges. Node color intensity and size were scaled according to degree centrality (DC) values, with higher DC values represented by larger node areas and darker hues. Using a two-step screening approach based on centrality metrics (BC, CC, and DC ≥ median values), core targets TP53, CASP3, MAPK3, and PTGS2 were identified (Figure 3B).

#### 3.7.2 GO enrichment analysis

Gene ontology (GO) enrichment analysis demonstrated that the intersecting target genes were significantly enriched in biological processes (BP) related to stress response and environmental adaptation, including: cellular responses to chemical stress, abiotic stimuli, and environmental stimuli; responses to reactive oxygen species (ROS), oxidative stress, metal ions, estradiol, and xenobiotic stimuli; and response to molecules of bacterial origin.

In terms of cellular component (CC), the analysis indicated that predominant enrichment of these genes in cytoskeletal motility structures (focal adhesion, pseudopodium), germ cell-specific compartments (germ cell nucleus), membrane signaling platforms (plasma membrane raft, membrane microdomain, caveola), immune granules (ficolin-1-rich granule and its lumen), and DNA replication machinery (replication fork).

Molecular function (MF) enrichment analysis revealed significant associations with the following three aspects: (i) ligand binding activities—organic acid binding, carboxylic acid binding, tetrapyrrole binding, heme binding, NADP binding, and FMN binding; (ii) oxidoreductase functions—monooxygenase activity and oxidoreductase activity acting on paired donors; (iii) apoptotic regulation—cysteine-type endopeptidase activity involved in apoptotic signaling pathways (Figure 3C).

#### 3.7.3 KEGG enrichment analysis

In order to systematically investigate the potential protective mechanism of 5-ALA on NOA, Kyoto encyclopedia of genes and genomes (KEGG) enrichment analysis was performed to predict the signaling pathways of potential targets of 5-ALA. As a result, 139 statistically significant pathways were identified, with key pathways including: apoptosis, p53 signaling, TNF signaling, AGE-RAGE signaling, VEGF signaling, relaxin signaling, lipid and atherosclerosis, and platinum drug resistance. Among them, the enrich factor for apoptosis is 15.67, with 8 closely related pathways, while the enrich factor for the p53 signaling pathway is 21.47, with 6 closely related pathways (Figures 3D,E).

### 3.8 Effects of 5-ALA/Fe^2+^ on the expression of specific proteins and apoptotic proteins in testes of NOA mice

**FIGURE 4:**
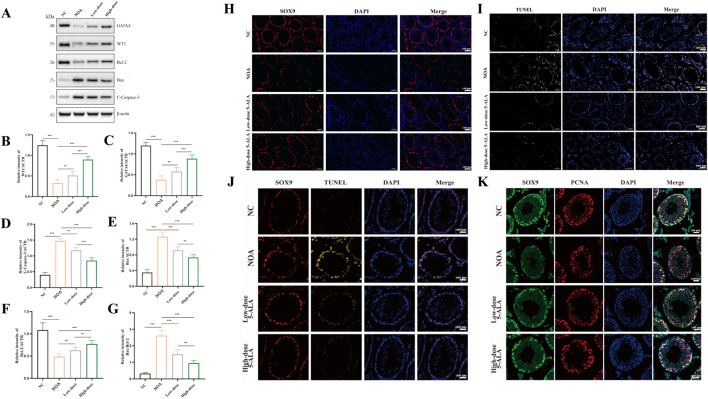
5-ALA/Fe^2+^restores Sertoli cell markers and protects Sertoli cells from apoptosis in NOA mice. **(A–G)** Expression of Sertoli cell-specific and apoptosis-related proteins in testicular tissues **(A)** Western blot bands. **(B–F)** Quantification of relative protein expression normalized to ACTB: **(B)** WT1, **(C)** GATA4, **(D)** Cleaved Caspase-3, **(E)** Bax, **(F)** Bcl-2. **(G)** Bax/Bcl-2 apoptotic ratio. **(H–K)** Immunofluorescence staining (scale bar = 100 μm). **(H)** Immunofluorescence staining of SOX9. **(I)** TUNEL staining. **(J)** Co-staining of SOX9 and TUNEL. **(K)** Co-staining of SOX9 and PCNA. Data are presented as mean ± SD (n = 10 in each group). *p < 0.05, **p < 0.01, ***p < 0.001 versus the indicated control group; ns, not significant.

The results of Western blot showed that compared with the normal control group, the expression of WT1, a marker protein that ensures normal cell differentiation and functional maintenance, was significantly downregulated in the testes of mice in the NOA group (P < 0.001). Notably, treatment with a low dose of 5-ALA/Fe^2+^ significantly increased WT1 expression (P < 0.01), and the upregulation was more pronounced in the high-dose group (P < 0.001) (Figures 4A,B). Similarly, another core protein GATA4, which regulate the normal development, differentiation and function of SCs, also showed reduced levels in NOA mice but significantly increased expression in the 5-ALA/Fe^2+^ treatment groups with statistically significant dose-dependent effects(P < 0.001) (Figures 4A,C). The results suggested that 5-ALA/Fe^2+^ ameliorated busulfan-induced impairments in the development, differentiation, and function of SCs in NOA mice.

To validate the predictions of network pharmacology and determine whether the protective effect was closely linked to apoptosis, we examined the protein expression of several key apoptotic molecules, including Bax, Bcl-2, and cleaved Caspase-3 (c-Caspase-3). The Western blot results demonstrated a marked dysregulation of apoptotic markers in NOA mice. Compared to the normal control group, the NOA group exhibited a significant increase in the expression of the pro-apoptotic protein Bax and the apoptosis executioner protein cleaved Caspase-3 (c-Caspase-3) (P < 0.001). 5-ALA/Fe^2+^ treatment effectively reduced the expression of both proteins, with the high-dose group showing a more pronounced effect than the low-dose group (P < 0.01) (Figures 4A,D,E). Conversely, the expression of the anti-apoptotic protein Bcl-2 was significantly decreased in NOA mice (P < 0.001). After treatment with 5-ALA/Fe^2+^, the expression of Bcl-2 was restored, and similarly, this upregulation was more significant in the high-dose group (P < 0.001) (Figures 4A,F). Critically, the Bax/Bcl-2 ratio, a gold-standard indicator of apoptotic propensity, was substantially increased in the testes of NOA mice (P < 0.001), while it markedly decreased through the treatment with 5-ALA/Fe^2+^. Similarly, the high-dose group showed a more pronounced downward trend than the low-dose group (P < 0.01) (Figures 4A,G).

### 3.9 Immunofluorescence analysis of Sertoli cell apoptosis and proliferation in testicular tissues

Compared to the control group, the intensity of SOX9 immunofluorescence signal in the testes of mice in the NOA model group was significantly reduced. In contrast, in the two groups treated with 5-ALA/Fe^2+^, the fluorescence signals were mainly distributed at the base of the seminiferous tubules near the tubular wall, which is consistent with normal histological structure. The fluorescence signal intensity increased with the dose of 5-ALA, showing a clear dose-dependent relationship (Figure 4H).

To evaluate apoptosis, TUNEL staining was performed. The NOA group exhibited a profoundly higher level of apoptosis compared to controls. Treatment with 5-ALA/Fe^2+^ led to a significant, dose-dependent amelioration of this effect, with the high-dose group showing the greater reduction in both the number of apoptotic cells and the associated fluorescence intensity (Figure 4I).

To further investigate the role of Sertoli cells and the effect of 5-ALA/Fe^2+^ on them, co-staining for SOX9 with TUNEL and for SOX9 with PCNA was performed on testicular sections from each group. In the normal control group, Sertoli cells exhibited normal morphology, with almost no TUNEL-positive cells and negligible co-localization with SOX9. Compared with the control group, the NOA model group showed a significantly increased proportion of TUNEL-positive cells among SOX9-positive Sertoli cells, indicating that Sertoli cells are one of the major cell types undergoing apoptosis in the busulfan-induced NOA model. Following treatment with different doses of 5-ALA/Fe^2+^, the proportion of TUNEL-positive Sertoli cells decreased and the co-localization signal was attenuated, suggesting that the treatment effectively inhibited Sertoli cell apoptosis (Figure 4J).

Conversely, SOX9/PCNA co-staining revealed opposite trends. In the NOA model group, the proportion of PCNA-positive cells among SOX9-positive Sertoli cells was significantly reduced, indicating that busulfan impaired the proliferative activity of Sertoli cells. After treatment with different doses of 5-ALA/Fe^2+^, the proportion of PCNA-positive Sertoli cells increased and the co-localization signal was enhanced, demonstrating that the treatment effectively promoted Sertoli cell proliferation (Figure 4K).

### 3.10 Characterization of isolated Sertoli cells

**FIGURE 5:**
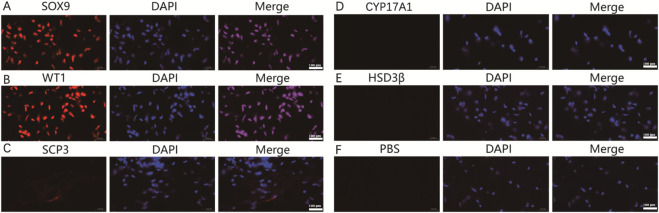
Identification of isolated mouse testicular Sertoli cells. Immunofluorescence staining showed that the isolated mouse testicular Sertoli cells expressed the Sertoli cell markers SOX9 **(A)** and WT1 **(B)**, but did not express the spermatogenic cell marker SCP3 **(C)** or the Leydig cell markers CYP17A1 **(D)** and HSD3β **(E)**. PBS **(F)** served as a negative control (scale bar = 100 μm). Purity of the isolated Sertoli cells was approximately 80%–90% as determined by WT1/SOX9 immunofluorescence counting (six random fields per coverslip).

Testicular Sertoli cells from mice in each group were isolated using a three-step enzymatic digestion method. After the Sertoli cells adhered to the culture surface, the spermatogenic cells in the supernatant were removed, and the medium was replaced with DMEM/F12 supplemented with 10% FBS. Freshly isolated Sertoli cells were identified using a panel of markers specific to testicular cell types. Immunocytochemical analysis revealed that the isolated cells expressed SOX9 and WT1 (Figures 5A,B), with strong fluorescence signals, whereas no specific fluorescence was observed in the negative control where PBS was used instead of the primary antibodies (Figure 5F). Immunofluorescence staining for the Leydig cell markers CYP17A1 and HSD3β, and spermatogenic cell marker SCP3 showed barely detectable signals (Figures 5C–E), indicating minimal contamination by other testicular cell types. Visual estimation (six random fields per coverslip) revealed that approximately 80%–90% of DAPI-positive nuclei expressed the Sertoli cell markers WT1 or SOX9 (Figure 5), confirming high purity suitable for subsequent experiments.

### 3.11 Flow cytometric analysis of apoptosis and mitochondrial membrane potential in cultured Sertoli cells

**FIGURE 6:**
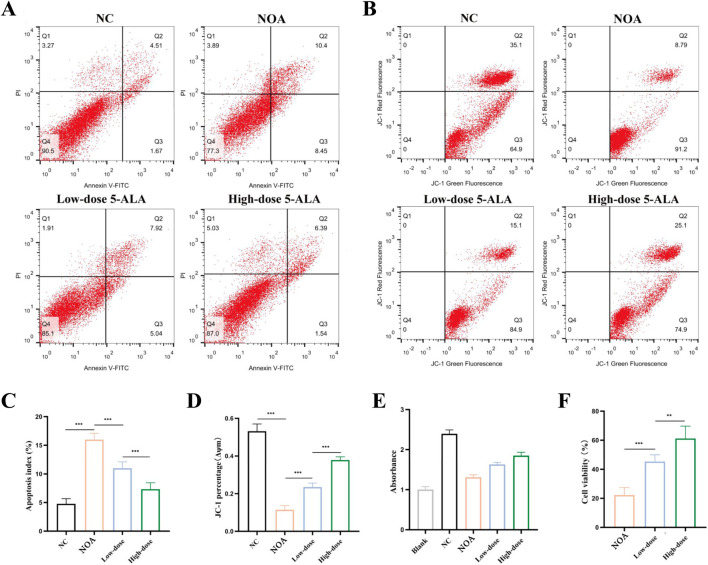
5-ALA/Fe^2+^ suppresses testicular cell apoptosis and enhances cellular viability in NOA mice. **(A,C)** Assessment of apoptosis by Annexin V-FITC flow cytometry. **(A)** Scatter plots. **(C)** Quantitative apoptosis index. **(B,D)** Analysis of mitochondrial membrane potential (ΔΨm) by JC-1 staining. **(B)** Flow cytometry plots. **(D)** Quantitative analysis of ΔΨm change. **(E,F)** Measurement of cell proliferation via CCK-8 assay. **(E)** Absorbance. **(F)** Proliferative activity. Data are presented as mean ± SD (n = 10 in each group). *p < 0.05, **p < 0.01, ***p < 0.001 versus the indicated control group; ns, not significant.

In order to quantitatively assess the apoptosis of SCs, we performed flow cytometry analysis with Annexin V-FITC and JC-1 staining. The Annexin V-FITC results revealed a significant increase in the apoptosis index in the NOA group (18.85%) compared to the normal control group (6.18%). 5-ALA/Fe^2+^ treatment reduced the apoptosis index to 12.96% in the low-dose group and to 7.93% in the high-dose group, which was close to the normal level. The differences among groups were statistically significant (P < 0.001) (Figures 6A,C).

JC-1 staining was used to monitor mitochondrial membrane potential (ΔΨm). The decrease in mitochondrial membrane potential indicated the activation of intrinsic apoptotic pathways in cells. Similarly, the percentage of cells with depolarized mitochondria was significantly higher in the NOA group (94.2%) than in the control group (64.9%). 5-ALA/Fe^2+^ treatment reduced this population to 84.9% and 74.9% in the low- and high-dose groups, respectively (Figure 6B). Correspondingly, compared with the normal control group, the average ΔΨm of cells in the NOA group was significantly reduced, suggesting severe mitochondrial dysfunction and activation of the intrinsic apoptotic pathway. The parameter was notably restored by 5-ALA/Fe^2+^ treatment, with a more pronounced recovery in the high-dose group. The differences among groups were statistically significant (P < 0.001) (Figure 6D).

### 3.12 Cell proliferation assessed by CCK-8 assay

The CCK-8 assay was performed to assess the viability of cells. compared with the normal control group, the NOA group demonstrated a significant decrease in absorbance, reflecting severe cell loss. Treatment with 5-ALA/Fe^2+^ resulted in a dose-dependent recovery of absorbance, with the high-dose group exhibiting a more pronounced increase than the low-dose group (Figure 6E). Accordingly, the viability of SCs was significantly impaired in the NOA group but was markedly restored by 5-ALA/Fe^2+^ treatment, with the high-dose group showing the more significant recovery. The differences among the groups were statistically significant (P < 0.001) (Figure 6F).

### 3.13 Proteomic analysis of the potential protective mechanism of 5-ALA/Fe^2+^

**FIGURE 7:**
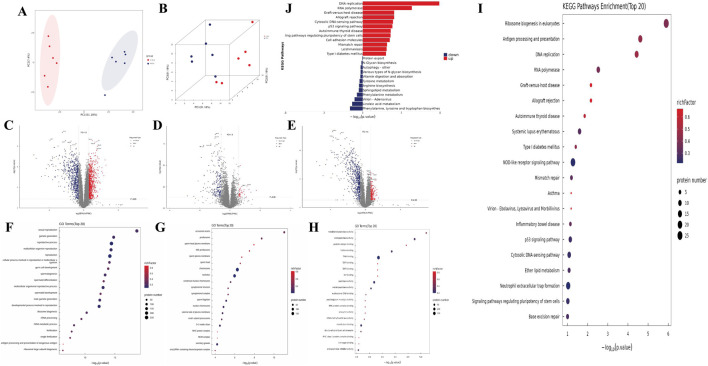
Proteomic analysis of the potential protective mechanism of 5-ALA/Fe^2+^ in NOA mice. **(A,B)** Principal component analysis (PCA) of the proteomic profiles: **(A)** 2D score plot, **(B)** 3D score plot. **(C–E)** Volcano plots of differentially expressed proteins (DEPs): **(C)** 5-ALA/Fe^2+^ treatment group vs. NOA model group, **(D)** NOA model group vs. control group, **(E)** 5-ALA/Fe^2+^ treatment group vs. control group. **(F–H)** Gene Ontology (GO) enrichment analysis of DEPs from the 5-ALA/Fe^2+^ vs. NOA model comparison: **(F)** Biological process (BP), **(G)** Cellular component (CC), **(H)** Molecular function (MF). **(I,J)** KEGG pathway enrichment analysis of DEPs (5-ALA/Fe^2+^ vs. NOA model): **(I)** Bubble plot of enriched pathways, **(J)** Butterfly plot displaying up- and downregulated pathways.

A proteomic analysis of SCs from each group was also conducted to validate the anti-apoptotic effect of 5-ALA/Fe^2+^. Principal Component Analysis (PCA) is an unsupervised data analysis method. It projects samples into a low-dimensional space based on the variance within the dataset. Samples with similar protein expression profiles tend to cluster closely, while those with substantial differences are positioned farther apart, thus visualizing both inter- and intra-group variability. In this study, two- and three-dimensional PCA was performed on all samples (Figures 7A,B). The results demonstrated strong cohesion within the control, NOA, and 5-ALA/Fe^2+^-treated groups, significant separation between groups, and high reproducibility of intra-group clustering. These outcomes confirmed that the data quality and group distinctions were suitable for subsequent analyses.

Using a significance threshold of fold change (FC) > 1.5 for upregulation or <0.67 for downregulation with P < 0.05, proteomic analysis identified 38 upregulated and 220 downregulated proteins in the NOA group versus normal controls, while comparison of 5-ALA-treated versus NOA groups revealed 779 upregulated and 709 downregulated proteins, with 4,816 total significantly altered proteins detected across all three experimental groups (Figures 7C–E).

#### 3.13.1 GO enrichment analysis

In order to gain a comprehensive and in-depth understanding of the functions, subcellular localization, and associated biological pathways of distinct protein groups in organisms, Gene Ontology (GO) was employed for protein annotation (Figures 7F–H).

GO enrichment analysis revealed that the testicular toxicity induced by busulfan in mice is mainly mediated through the disruption of DNA-related biological processes, as well as the impairment of antigen processing, presentation, and immunogenic functions, thereby compromising cellular immune responses.

Compared to the NOA group, the differentially expressed proteins in the 5-ALA/Fe^2+^-treated group were primarily enriched in the processes associated with germ cell growth and development, reproduction, and immune inflammation.

A comparative analysis of the NOA group, treatment group, and normal control group revealed that the most significant differences among these groups were primarily associated with RNA-related pathways, along with nucleic acid metabolism, protein folding, and reproduction-related processes. These findings suggest that the intergroup interactions likely focus on RNA transcription, protein biosynthesis, organismal metabolism, and reproductive functions.

Based on the above analysis and the alleviation of symptoms in NOA mice, it further indicates that 5-ALA/Fe^2+^ has a significant protective effect on SCs in the testes.

#### 3.13.2 KEGG enrichment analysis

To systematically and comprehensively investigate biological processes, disease pathology, drug mechanisms, and so on, it is often essential to interpret phenotypic changes from the perspective of a series of protein coordination effects—such as alterations in metabolic pathways. Therefore, protein annotation and analysis were conducted using the KEGG database.

All significantly differentially expressed proteins were mapped to the KEGG database for functional annotation. Fisher’s exact test was then applied to evaluate the statistical significance of pathway enrichment among these proteins. Pathways with a corrected P-value of less than 0.05 were considered significantly enriched.

KEGG enrichment analysis revealed that, compared to the NOA group, the differentially expressed proteins in the 5-ALA/Fe^2+^-treated groups were significantly enriched in the following pathways: ribosome biogenesis in eukaryotes, antigen processing and presentation, DNA replication, RNA polymerase, graft-versus-host disease, allograft rejection, autoimmune thyroid disease, systemic lupus erythematosus, type I diabetes mellitus, NOD-like receptor signaling pathway, mismatch repair, asthma, infection by Ebolavirus, Lyssavirus, and Morbillivirus, inflammatory bowel disease, p53 signaling pathway, cytosolic DNA-sensing pathway, ether lipid metabolism, neutrophil extracellular trap formation, signaling pathways regulating pluripotency of stem cells, and base excision repair (Figure 7I).

To further evaluate the significance of pathway enrichment among differentially expressed proteins, KEGG enrichment analysis on the up- and downregulated proteins was performed separately. The results revealed that pathways associated with DNA synthesis and repair, inflammatory and immune responses, apoptosis, and signal transduction were significantly upregulated. In contrast, pathways related to partial amino acid metabolism, lipid metabolism, protein transport, and autophagy were notably downregulated (Figure 7J).

Taken together, the proteomic findings further support that TP53/CASP3-related apoptotic signaling likely plays a critical role in the ameliorative effects of 5-ALA/Fe^2+^ on testicular damage in NOA mice. Additionally, 5-ALA/Fe^2+^ may exert multifaceted protective actions by promoting DNA repair, improving immune function, and reversing disrupted metabolism.

### 3.14 Protein-level validation of predicted targets

**FIGURE 8:**
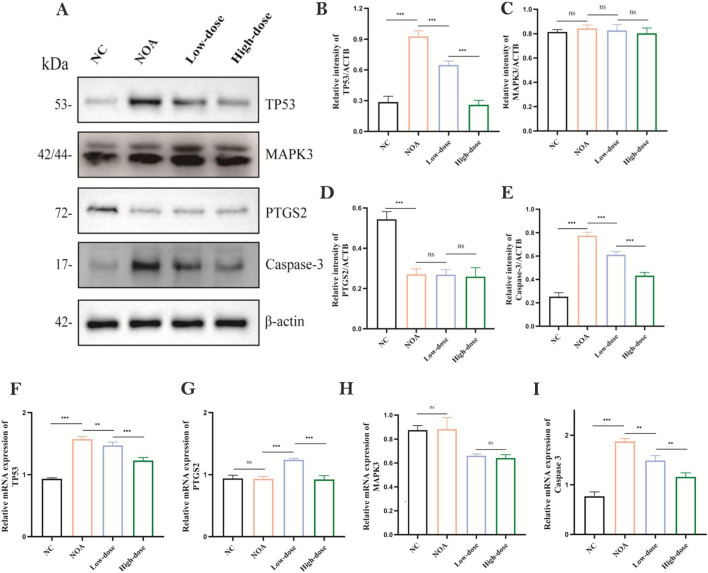
Experimental validation of core targets predicted by network pharmacology. **(A–E)** Western blot analysis of core target proteins. **(A)** Representative Western blot images. **(B–E)** Quantitative analysis of protein expression levels normalized to ACTB: **(B)** TP53, **(C)** MAPK3, **(D)** PTGS2, **(E)** Caspase-3. **(F–I)** qPCR analysis of mRNA expression for the corresponding targets. **(F)** TP53, **(G)** MAPK3, **(G)** PTGS2, **(H)** MAPK3, **(I)** Caspase-3. Data are presented as mean ± SD (n = 10 in each group). *p < 0.05, **p < 0.01, ***p < 0.001 versus the indicated control group; ns, not significant.

The targets predicted by the above network pharmacology include TP53, CASP3, MAPK3, and PTGS2. The expression levels of these target molecules in the testes of mice were detected by Western blot. The results revealed that the expression of TP53 and CASP3 showed the same trend and were both significantly increased in the NOA mice compared to the control. 5-ALA/Fe^2+^ treatment reversed this increase, with a more pronounced reduction observed in the high-dose group. The difference among groups was statistically significant (P < 0.001) (Figures 8A,B,E).

However, MAPK3 expression remained stable across all groups, showing no significant alterations (Figures 8A,C). Notably, PTGS2 expression was substantially downregulated in both the NOA and treatment groups compared to the control (P < 0.001) and there was no significant difference between the disease model and the therapeutic groups (Figures 8A,D).

### 3.15 mRNA-level validation of predicted targets

The mRNA expression levels of these targets were subsequently analyzed by qPCR. The mRNA results of TP53 and CASP3 showed a trend consistent with the results of Western blot, upregulated in NOA, but decreased after treatment with 5-ALA/Fe^2+^, and the effect of high-dose 5-ALA was better than that of low-dose group (P < 0.001) (Figures 8F,I).

For PTGS2, its mRNA level was relatively higher only in the low dose group compared to the NOA group, with no other significant differences observed among the other groups (Figure 8G). Similarly, the mRNA expression of MAPK3 showed no significant difference between the control and NOA groups. However, unlike the stable protein level, MAPK3 transcription was downregulated in both 5-ALA/Fe^2+^ treatment groups compared to the control, but there was also no significant difference between the two groups (Figure 8H).

## 4 Discussion

Male infertility has emerged as a critical global health issue, with NOA-induced infertility being one of the most severe cases, accounting for 15%–21% of all male infertility cases. Unfortunately, the underlying mechanisms of NOA remain poorly understood, leading to a lack of standardized and effective treatment strategies in clinical practice.

In this study, we established a NOA mouse model via intraperitoneal injection of busulfan. Subsequently, 5-ALA/Fe^2+^ medication was administered to investigate its promoting effect on the recovery of spermatogenic function in NOA mice. Collectively, the results of the present study demonstrated that 5-ALA/Fe^2+^ administration effectively reversed busulfan-induced damage in testicular and epididymal tissues, improved morphological and functional parameters in NOA mice, ameliorated hormonal imbalance and suppressed cellular apoptosis to help restore reproductive function. Furthermore, the high-dose treatment was more effective than the low-dose regimen, indicating a dose-dependent therapeutic effect.

From the perspective of histopathology, the results of this study showed that busulfan caused significant atrophy of the testicular morphology, with pathological manifestations including large areas of degeneration and necrosis of germ cells in the testes, significant atrophy of the seminiferous tubules, and reduction of interstitial cells. In addition, inflammatory cell infiltration can be observed in the interstitial tissue of the testes. Critically, the absence of sperm in the seminiferous tubules and epididymis provides a direct pathological basis for the infertile phenotype. After treatment with 5-ALA, the pathological manifestations of NOA were significantly improved. The most immediate observation was a macroscopic amelioration of testicular atrophy. The seminiferous tubules displayed an improved structural integrity, characterized by a notable thickening of the germinal epithelium, restoration of its cellular layers, and a marked increase in the population of germ cells. Consequently, this led to a significant rise in sperm count within the epididymis.

A notable observation was the significant downregulation of the Sertoli cell marker SOX9 in the NOA group, even though the basic architecture of the seminiferous tubules appeared preserved in the results of HE staining. This paradox suggests that in the busulfan-induced NOA model, Sertoli cells, while not completely disappeared, may suffer from severe functional damage similar to germ cells, leading to reduced expression of their specific markers. Treatment with 5-ALA/Fe^2+^ facilitated the recovery of normal Sertoli cell phenotype and function, as evidenced by the restoration of SOX9 signal. This finding highlights the therapeutic significance of 5-ALA/Fe^2+^ in protecting and restoring Sertoli cell function, beyond merely supporting their survival.

In terms of biochemical indicators, it has been reported that most NOA patients have the disturbance of various hormones, comprehensively manifesting as a triad of elevated LH and FSH, and low testosterone (Li et al., 2024a). The hypothalamic-pituitary-testicular (HPT) axis is pivotal to this system. Specific hypothalamic nuclei synthesize gonadotropin-releasing hormone (GnRH). This subsequently stimulates gonadotropes in the anterior pituitary to synthesize and release luteinizing hormone (LH) and follicle-stimulating hormone (FSH). Typically, LH acts directly on testicular Leydig cells to control their steroidogenic activity, stimulating the production of testosterone (Holota et al., 2020). Correspondingly, sex steroids exert a central negative feedback effect on their own synthesis. Meanwhile, FSH supports spermatogenesis via its action on Sertoli cells, which secrete inhibin B(INHB) to provide specific negative feedback on pituitary FSH release (Leslie et al., 2023).

Consistent with previous studies (Rostami et al., 2022; Ryu et al., 2023; Raffo et al., 2025), our study demonstrated a significant decrease in testosterone and INHB, alongside a marked increase in LH in the busulfan-induced NOA mice. The decrease of testosterone is suggestive of Leydig cell dysfunction and is also associated with germ cell depletion, accompanied by a compensatory increase in LH. The pathophysiological basis for the characteristic decline in INHB is the profound depletion of functional Sertoli cells. Additionally, the thickened seminiferous tubular wall likely compromises the integrity of the blood-testis barrier, potentially altering hormone diffusion and contributing to the aberrant peripheral hormone profiles (Raffo et al., 2025). And the 5-ALA/Fe^2+^-mediated restoration of testicular structure and function appeared to enhance testosterone secretion and seminiferous tubule volume, as well as improve the function of supporting cells to restore INHB levels.

Beyond the aforementioned phenotypes, our findings demonstrate that the therapeutic effect of 5-ALA/Fe^2+^ critically involves the specific protection of SCs from apoptosis. First, TUNEL staining revealed that busulfan induced overall testicular cell apoptosis, which was substantially alleviated by 5-ALA/Fe^2+^.Furthermore, co-staining of SOX9 with TUNEL and PCNA directly confirmed that 5-ALA/Fe^2+^ effectively inhibited apoptosis and promoted the proliferative activity of SCs. Second, the balance of key apoptotic regulators was restored. The pro-apoptotic protein Bax and the executioner protein c-caspase-3, both upregulated due to cytotoxicity of busulfan, were downregulated following intervention of 5-ALA/Fe^2+^. Conversely, the suppressed expression of the anti-apoptotic protein Bcl-2 in NOA group was reversed. The shift indicates a re-establishment of the apoptotic homeostasis. Critically, experiments on isolated and purified SCs provided direct evidence. Direct assessment of these purified cells revealed that 5-ALA/Fe^2+^ treatment improved mitochondrial membrane potential of SCs, and enhanced cell proliferation. Given the germ cell-depleted context of the model, the concordant anti-apoptotic effects at multiple levels indicate that SCs are a primary target of 5-ALA/Fe^2+^.Therefore, it can be reasoned that 5-ALA/Fe^2+^ exerts its beneficial effects substantially by mitigating apoptosis in SCs, thereby contributing to the preservation of the testicular microenvironment.

SCs serve as essential coordinators of spermatogenesis and are key determinants of testicular size and function (O'Donnell et al., 2022). They direct testicular differentiation, promote testicular growth, support germ cell development and sperm release, drive Leydig cell function and development, and influence other somatic cell functions—including peritubular cell differentiation, vascular maintenance, and immune privilege (Wang et al., 2022; Li et al., 2024b; Zhao et al., 2020). More importantly, SCs are far more than passive supporting cells; they are now recognized as central architects and regulators of the spermatogonial stem cell (SSC) niche. By secreting critical signaling molecules such as GDNF and FGF2, and by forming the blood–testis barrier, they precisely balance SSC self-renewal and differentiation, thereby governing the homeostasis of the spermatogenic microenvironment (Liu et al., 2024).

In the present study, 5-ALA/Fe^2+^ treatment significantly upregulated GATA4 and WT1 expression in the testes of NOA mice. Previous studies have reported that downregulation of the transcription factor WT1 in Sertoli cells accelerates cellular senescence, disrupts tight junctions, and impairs cell identity (Huang et al., 2023; Lee et al., 2023; Gao et al., 2024). These alterations compromise Sertoli cell homeostasis, creating a dysfunctional microenvironment that inhibits spermatogenesis and is closely linked to the depletion of the SSC pool.

GATA-binding protein 4 (GATA4) is a key transcription factor involved in maintaining the SSC pool. Rubicon functions as a negative regulator of autophagy in Sertoli cells, preserving normal cellular function by preventing autophagic degradation of GATA4, thereby supporting SSC pool maintenance (Gao et al., 2024). Moreover, GATA4 has been shown to bind and regulate Sox9 expression, contributing to testicular differentiation (Ogawa et al., 2025).

These molecular changes indicate that 5-ALA/Fe^2+^ not only protects Sertoli cells through anti-apoptotic effects but also enhances their paracrine function, thereby promoting Leydig cell regeneration and functional recovery, and systematically improving the testicular spermatogenic microenvironment. Thus, the protective role of 5-ALA/Fe^2+^ in Sertoli cells extends far beyond cell survival. It facilitates the repair of the damaged SSC niche, provides essential support signals for residual SSCs, and ultimately initiates the slow but synergistic reconstruction of spermatogenesis.

The network pharmacology predictions indicated that busulfan-induced reproductive toxicity primarily acts through DNA damage-triggered apoptosis of germ cells and oxidative stress-mediated disruption of the blood-testis barrier and testicular microenvironment. Conversely, 5-ALA could reverse busulfan-induced testicular injury caused by regulating multiple biological processes, including apoptosis suppression, oxidative stress alleviation, immune response modulation, angiogenesis promotion, and lipid metabolism normalization. Notably, TP53 and CASP3 were identified as key targets within the apoptotic signaling network, and subsequent experimental validation confirmed a pivotal role of this TP53/CASP3-mediated apoptotic pathway in the therapeutic mechanism of 5-ALA.

However, although other key targets such as MAPK3 and PTGS2 were also predicted by network pharmacology, the follow-up experiments revealed that neither their protein nor mRNA expression levels exhibited a consistent therapeutic trend in response to 5-ALA treatment. Several factors may account for this discrepancy. First, PTGS2 expression in the testis is largely restricted to macrophages and Leydig cells; our whole-tissue Western blotting mixed all cell types, and in the busulfan-induced NOA model, Leydig cells were markedly reduced, leading to low PTGS2 levels across groups. Thus, cell-type restricted expression explains the lack of detectable changes. Second, MAPK3 is primarily regulated by post-translational phosphorylation, whereas conventional Western blotting measures total protein, which often does not change substantially; therefore, our negative results do not rule out pathway involvement, and future phosphorylation analysis is needed. Third, ligand-based network pharmacology is inherently noisy with a tendency for false-positive predictions, so it is not uncommon that predicted targets fail experimental validation. Nevertheless, due to this lack of concordance between expression levels and the anticipated therapeutic effect, MAPK3 and PTGS2 were excluded from our final mechanistic model, as their involvement appears to be less direct.

The proteomic analysis provided independent and compelling evidence to consolidate the proposed mechanism. Notably, significant upregulation of the p53 signaling pathway was observed, indicating that TP53/CASP3-mediated apoptosis is a key mechanism by which 5-ALA/Fe^2+^ alleviates testicular injury in NOA mice. It should be noted that the HO-1/Nrf2 axis, which has been shown to mediate the protective effects of 5-ALA/Fe^2+^ in other contexts including our prior work on testicular heat stress, was not examined in the present study, as we deliberately focused on the TP53-CASP3 apoptotic node; this axis will be systematically investigated in our follow-up studies. The parallel reduction of multiple apoptotic and inflammatory markers is expected upon inhibition of the TP53-CASP3 axis, as this pathway orchestrates a broad downstream transcriptional and proteolytic cascade. Beyond this core finding, the proteomic information also suggested that 5-ALA/Fe^2+^ may exert broader, multifaceted protective effects. These potential benefits, aligned with the network pharmacology predictions, appear to involve promoting DNA repair processes, modulating immune function, and reversing the busulfan-disrupted metabolic landscape, synergistically contributing to the restoration of testicular homeostasis.

In the present study, the ratio of 5-ALA/Fe^2+^ was selected based on preliminary experiments, which identified two effective dose combinations with no apparent toxicity. Our preliminary results showed that 30 mg/kg and 100 mg/kg were effective, whereas 10 mg/kg had no significant effect, and 300 mg/kg did not provide additional benefit compared to 100 mg/kg (Hou et al., 2013; Hou et al., 2015; Gao et al., 2023). Therefore, these two doses were used in the subsequent experiments.

No acute systemic toxicity associated with 5-ALA/Fe^2+^ was observed during the 4-week treatment period. However, given its well-documented photosensitizing properties, future studies should comprehensively evaluate potential long-term risks, particularly chronic phototoxicity and sustained effects on metabolic pathways. Furthermore, iron plays a dual role *in vivo*. As an essential cofactor in heme biosynthesis, appropriate levels of Fe^2+^ facilitate the conversion of 5-ALA to heme and the subsequent generation of antioxidant heme proteins; conversely, excess free Fe^2+^ may exacerbate oxidative injury by catalyzing the Fenton reaction, leading to accumulation of reactive oxygen species (ROS) and subsequent ferroptosis (Obi et al., 2022). The intracellular accumulation of protoporphyrin IX (PpIX) is regulated by complex mechanisms intimately linked to heme metabolism and cellular iron homeostasis. 5-ALA is a key intermediate in the heme biosynthetic pathway, and heme oxygenase-1 (HO-1) is the rate-limiting enzyme for heme degradation, catalyzing the production of free iron, carbon monoxide, and biliverdin/bilirubin (Hou et al., 2013; Nishio et al., 2014; Hou et al., 2015). While elevated HO-1 expression in normal cells primarily represents an adaptive antioxidant response aimed at mitigating oxidative stress, excessive release of free Fe^2+^—particularly when cellular iron storage or chelation capacity (e.g., ferritin saturation) is overwhelmed—can massively trigger the Fenton reaction, leading to ROS accumulation and subsequent ferroptosis (Li and Ogura, 2025; Pustimbara et al., 2024).

In light of this dual role of iron, we further evaluated oxidative stress levels (SOD and MDA) in each group of mice. The results demonstrated that at both 30 mg/kg and 100 mg/kg, 5-ALA/Fe^2+^ exerted therapeutic effects, effectively alleviating busulfan-induced testicular oxidative stress. The treatment showed a favorable safety profile within the dose range used in this study, and its protective mechanism may involve enhancing antioxidant capacity and inhibiting lipid peroxidation.

Collectively, the present study delineated a compelling mechanism through which 5-ALA/Fe^2+^ reversed busulfan-induced testicular injury, primarily by restoring the Bax/Bcl-2 balance and inhibiting caspase-3 activation, thereby suppressing excessive apoptosis in a dose-dependent manner. The main innovations of this study are threefold. Firstly, in terms of treatment strategy, we have taken the lead in exploring the application of 5-ALA in NOA therapy, providing a new potential solution to the clinical management of this challenging disease. Secondly, at the mechanistic level, this study elucidates for the first time a key mechanism by which 5-ALA primarily restores testicular spermatogenic function by regulating the TP53-CASP3 apoptotic signaling axis to improve supporting cell function. The complete signaling pathway has not been reported before in the field of NOA. Finally, from the perspective of clinical significance, the study indicated that faced with NOA, drug intervention targeting the apoptotic pathway is probably a feasible strategy with significant translational value.

While this study proposes a potentially promising therapeutic strategy, several limitations must be acknowledged. First, although the safety of 5-ALA was preliminarily evaluated, the 4-week treatment period is insufficient to assess chronic toxicity or long-term off-target effects; longer-term studies are needed. Moreover, we did not examine the effect of 5-ALA/Fe^2+^ in healthy mice, so its safety under physiological conditions remains to be confirmed. Second, the lack of systematic evaluation of redox status at both the testicular tissue and cellular levels restricts our ability to precisely delineate the balance between antioxidant and pro-oxidant effects under the present dosing regimen. Third, the specific molecular initiator—whether upstream or downstream of the TP53-CASP3 axis—through which 5-ALA exerts its regulatory effects remains to be identified. Fourth, despite multiple attempts, serum FSH levels could not be successfully measured in this study; optimization of FSH assay conditions is required, and future studies should address this. Fifth, no protein-level FDR correction was applied in our proteomic analysis; therefore, the numbers of up- and downregulated proteins may contain false positives. Nonetheless, the key conclusions were validated by orthogonal methods including Western blotting and qPCR. Sixth, we acknowledge that the busulfan-induced testicular injury model, while useful for studying chemotherapy-related spermatogenic damage, does not fully represent the clinical heterogeneity of human NOA. Specifically, this model recapitulates germ cell depletion but does not reproduce the full spectrum of human NOA subtypes, including Sertoli cell-only syndrome, maturation arrest, as well as idiopathic, genetic, and inflammatory forms. Moreover, the pharmacokinetics of oral 5-ALA/Fe^2+^ in humans may differ from that in mice. Future investigations will focus on further elucidating the mechanistic basis of 5-ALA/Fe^2+^ combination, acquiring long-term safety data, and evaluating its therapeutic potential in clinical settings.

## 5 Conclusion

The study definitively identifies 5-ALA/Fe^2+^ as a potent protector against busulfan-induced testicular injury and spermatogenic failure. The data pinpoint the suppression of the TP53/CASP3-mediated apoptotic pathway as a key downstream node, which is manifested as significant recovery of both testicular structure and function, with the high-dose regimen yielding superior therapeutic outcomes. The study provides critical initial evidence for the potential clinical application of 5-ALA/Fe^2+^. Therefore, the drug represents a promising candidate for further development as a therapeutic strategy for NOA and other forms of male infertility driven by aberrant apoptosis.

## Data Availability

The raw data supporting the conclusions of this article will be made available by the authors, without undue reservation.
